# Organizational Meeting Orientation: Setting the Stage for Team Success or Failure Over Time

**DOI:** 10.3389/fpsyg.2019.00812

**Published:** 2019-04-17

**Authors:** Joseph E. Mroz, Nicole Landowski, Joseph Andrew Allen, Cheryl Fernandez

**Affiliations:** ^1^Denison Consulting, Ann Arbor, MI, United States; ^2^Department of Psychology, University of Nebraska Omaha, Omaha, NE, United States; ^3^Gallup Inc., Omaha, NE, United States

**Keywords:** meetings, groups, teams, job attitudes, time

## Abstract

Teams are an integral tool for collaboration and they are often embedded in a larger organization that has its own mission, values, and orientations. Specifically, organizations can be oriented toward a variety of values: learning, customer service, and even meetings. This paper explores a new and novel construct, organizational meeting orientation (the set of policies and procedures that promote or lead to meetings), and its relationship to perceived team meeting outcomes and work attitudes. An organization’s policies, procedures, and overall orientation toward the use of team meetings—along with the quality and perceived effectiveness of those meetings—set the stage for how teams develop and collaborate. Across two exploratory studies, we demonstrate that perceptions of an organization’s orientation toward meetings is associated with the perceived quality and satisfaction of team meetings, along with work engagement and intentions to quit. Employees who feel meetings lack purpose or are overused tend to be less engaged with their work and more likely to consider leaving the organization. Based on the findings, we conclude with a robust discussion of how meeting orientation may set the stage for team interactions, influencing how their team operates over time on a given project or series of projects. An organization’s orientation toward meetings is a new construct that may exert an influence on team dynamics at *the organizational level*, representing a factor of the organization that affects how and when teams meet and collaborate.

## Introduction

Workplace meetings are essential to both the functioning of organizations and employees’ workplace experiences. Of the estimated 55 million meetings occurring daily in the United States, managers in large organizations are dedicating over three-quarters of their time preparing for, attending, leading, and processing meeting results (Keith, 2015). Among the various reasons to call a meeting, workplace meetings can be used to share information (McComas, 2003), brainstorm (Reinig and Shin, 2002), socialize (Horan, 2002), and solve problems (e.g., McComas et al., 2007). Being that meetings are an integral part of organizations, firms may have a unique culture of policies, procedures, and practices that promote, emphasize, and result in meetings – that is, a meeting orientation (Hansen and Allen, 2015). Meeting orientation is a relatively unexplored topic in meeting science, and no empirical studies have looked at its relationship to employee attitudes concerning meetings or their broader work environments (Allen and Hansen, 2011; Hansen and Allen, 2015). An organization’s overall culture toward meetings (i.e., meeting orientation) may have important consequences for how groups and teams develop over time by, for instance, influencing how often, when, and under what circumstances group members come together to work and discuss problems.

Across two studies, we propose that there are a number of ways in which individuals’ belief about the meeting orientation of their organization may influence how people view various meeting and organizational outcomes, which can subsequently influence team development over time. Specifically, building upon the original theory and conceptualization by Hansen and Allen (2015), we argue that meeting orientation is related to employees’ satisfaction with meetings and the perceived effectiveness of meetings, along with broader work-related attitudes such as intentions to quit (ITQ) and work engagement. Consistent with other theories of and empirical evidence for organizational orientations (e.g., market orientation; Kirca et al., 2005), we believe meeting orientation will relate to both proximal (team meeting satisfaction) and distal (work engagement) individual outcomes. After establishing meeting orientation as an important construct of interest in meeting science and for organizations, we provide a discussion and testable propositions for future research regarding how meeting orientation, and a firm’s overall cultural toward meetings, can influence how teams develop and grow over time.

### Organizational Orientations and the Meeting Orientation

Organizational orientations provide a potential competitive advantage for firms and examples include a market orientation or entrepreneurial orientation (Kirca et al., 2005; Rauch et al., 2009). A particularly relevant organizational characteristic that may affect team meeting processes and outcomes, as well as employee attitudes toward the organization, is an organization’s meeting orientation, or the policies, procedures, and practices that emphasize, promote, or leads to meetings (Hansen and Allen, 2015). As market, entrepreneurial, and learning orientations affect how an organization structures itself and operates (e.g., Matsuno et al., 2005), a meeting orientation describes the value that an organization places on meetings (i.e., team meetings) and how often meetings are used as a collaborative tool. The meeting orientation serves as the mode by which other organizational orientations permeate and are enacted across the organization. That is, unlike other organizational orientations, meeting orientation is a process focused orientation specific to how people in the organization interact with one another in, through, and around their group and team meetings.

The degree to which an organization is oriented toward the use of group and team meetings is best represented on a continuum from low to high (Hansen and Allen, 2015). Organizations with a high meeting orientation implicitly or explicitly encourage employees to use group and team meetings as an important form of interaction and the overall work process. Therefore, high meeting orientation organizations may hold many workplace meetings, but those group and team meetings are not necessarily *good* meetings. Likewise, low meeting orientation organizations may hold fewer meetings, and meetings are not necessarily higher or lower quality than in organizations with a different meeting orientation. For example, meetings may be viewed negatively when a meeting culture inhibits employees from doing their job because they attend too many group and team meetings. Alternatively, additional meetings that provide employees the opportunity to pose questions to executive management can be viewed positively (Hansen and Allen, 2015). Depending on the context, these meeting cultures may be advantageous or disadvantageous.

Meeting orientation is composed of four facets: policy focus, rewards for meetings, strategic use of meetings, and overuse of meetings (Hansen and Allen, 2015). Policy focus refers to the strength of formal policies and procedures at the organizational level with respect to meetings. Rewards for meeting speaks to how much organizational members believe that the organization rewards people who attend, lead, or organize meetings. Strategic use of meetings deals with how much an organization relies on meetings to gather, disseminate, or respond to information. Finally, meeting overuse refers to how much an organization utilizes meetings too often or holds meetings that are too long.

Despite the potential relevance and impact that an organization’s meeting orientation may have on the way employees interact, no published research has empirically evaluated the relation between meeting orientation and meeting outcomes. As previously mentioned, a high or low meeting orientation does not necessarily provide an indication as to the quality of an organization’s meetings or how satisfied employees are with their group and team meetings at work. However, based on the nature of several meeting orientation facets, there are a number of ways in which individuals’ beliefs about the meeting orientation of their organizations may influence how people view their meetings. Further it may influence how they view their organization and it may enable or constrain their team’s ability to function over time.

### Overview of Studies

We conducted two studies to investigate the concept of meeting orientation and its relation to team meeting and organizational outcomes. These were exploratory studies designed to be a “first look” at the concept of a meeting orientation and how it may be related to organizationally relevant employee attitudes. Our first study sought to explore whether policy focus, rewards, strategic usage, and potential overuse were advantageous or disadvantageous to perceptions of team meeting quality. Given that meetings are events that can be strategically used to foster employee engagement (Allen and Rogelberg, 2013), in Study 2 we explored whether the facets of meeting orientation were related to work-related outcomes such as employee engagement and ITQ.

## Study 1

The four facets of meeting orientation will likely differentially relate to team meeting outcomes. First, one facet of meeting orientation is group and team meeting overuse, or how much an organizational member thinks that the organization has too many meetings, has meetings that are too long, or routinely holds meetings just because meetings are scheduled. Individuals who believe that their organization overuses group and team meetings are likely to think that, in general, meetings are not effective or satisfying. One aspect of an effective meeting is having and achieving goals. Routine or “standing” meetings, and other meetings generally, may have no clear goals, making it difficult for the meeting to be effective. Likewise, people tend to dislike meetings (Tracy and Dimock, 2004), and this dislike may intensify if individuals believe that their organizations have too many meetings. Finally, people may not trust their group or team meeting leader’s managerial abilities or capacity to “do the right thing” if meeting attendees think the organization has too many meetings. Employees may view managers, who typically lead team meetings at work, as embodiments of the organization (Eisenberger et al., 1986), and if the organization overuses meetings, then the manager overuses group and team meetings. Therefore, we hypothesize the following:

Hypothesis 1: Overuse will be negatively related to team meeting effectiveness (1a) and team meeting satisfaction (1b).

The other three facets should have a markedly different relationship to meeting outcomes. Strategic use of meetings, or how much meeting attendees believe their organizations use group and team meetings to gather, exchange, and act on information, may be positively related with both team meeting effectiveness and team meeting satisfaction. People who believe that their organizations have meetings for a purpose, namely to interact with information, are likely to believe that those group and team meetings are effective and satisfying because the purpose is readily apparent and aligns with important, widely held assumptions about what a work meeting should be (Allen et al., 2014).

Policy focus and rewards may also influence how supported group and team meeting attendees feel from the organization. Support in this case derives from perceived organizational support (POS) theory (Eisenberger et al., 1986), which refers to the extent to which employees believe that their work organization cares about their wellbeing and values their contribution. A team meeting leader is supportive by valuing contributions of attendees and by fostering a caring atmosphere in their group or team meetings. If an organization has an orientation toward the strategic use of meetings and the organization rewards the use of meetings, team meeting attendees may feel that the meeting leader is supportive. For instance, if a meeting has a purpose for information sharing and the organization encourages these sorts of group and team meetings, meeting leaders may become adept at conducting these meetings by supporting and encouraging the participation of all attendees. Likewise, if group and team meetings are overused and lack purpose, attendees may not feel supported because their meeting role is unclear or the meeting is generally unnecessary.

Hypothesis 2: Policy focus (2a), rewards (2b), and strategic use of meetings (2c) will be positively related to team meeting satisfaction.
Hypothesis 3: Policy focus (3a), rewards (3b), and strategic use of meetings (3c) will be positively related to team meeting effectiveness.

**Figure 1:**
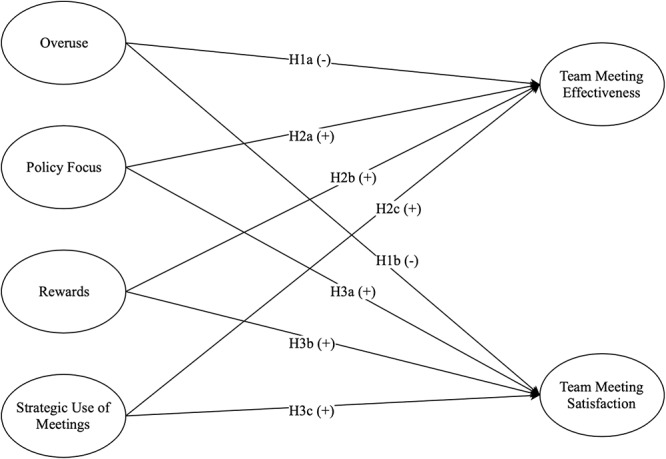
Hypothesized relationships in Study 1.

Figure 1 includes hypothesized relationships in Study 1.

### Methods

#### Participants and Procedure

In exchange for course credit, students in an undergraduate psychology course recruited working adults to participate in the study through Qualtrics, an online survey tool. A total of 22 students sent invitations to potential participants, 174 of whom finished the survey. Thus, the final sample consisted of 174 well-educated adults (59% held a four-year degree) who ranged from 19 to 68 years old (*M* = 38.72, *SD* = 13.03). Of participants who provided information, 30% were men. Respondents worked in a variety of industries such as healthcare, education, and the military. Workers who supervised at least one employee comprised 48% of the sample.

Due to the cross-sectional nature of the design, we implemented several procedures to mitigate concerns of common method bias (Podsakoff et al., 2003). Adhering to the recommendations proposed by Podsakoff et al. (2003), which are aimed at reducing demand characteristics and evaluation apprehension, participants were assured that they would be provided with anonymity, and that their responses would not be considered right or wrong. We also followed recommendations suggested by Conway and Lance (2010), which include utilizing counterbalancing of measures and demonstrating adequate evidence of measure reliability. In an effort to mitigate concerns of item-context-induced mood states, priming effects, and biases related to the order of measures or individual items, all measures and items were counterbalanced via randomization. Furthermore, each item utilized simple and precise language, addressing one particular concept, as suggested by Tourangeau et al. (2000).

#### Measures

##### Team meeting effectiveness

Participants indicated how effective they felt their last meeting was across six areas (e.g., “Achieving your own work goals” and “Providing you with an opportunity to acquire useful information”) using a 5-point Likert scale (1 = *very ineffective*; 5 = *very effective*). Cronbach’s alpha for this measure was 0.83.

##### Team meeting satisfaction

Meeting satisfaction was measured using a 6-item measure developed by Rogelberg et al. (2010). Participants read a question stem (“My last meeting was…”) followed by series of adjectives and indicated how well each one described their last meeting (e.g., “stimulating” and “boring”) from 1 (*strongly disagree*) to 5 (*strongly agree*). Cronbach’s alpha estimate of internal consistency was 0.85.

##### Meeting orientation

Allen and Hansen’s (2011) meeting orientation scale consists of four facets: policy focus, rewards, strategic use, and overuse. Three items comprise each facet. Participants indicated their agreement or disagreement to statements for each facet. Items for policy focus included my firm “has policies that promote meetings,” “has a lot of standard procedures associated with meetings,” and “has what could be called a meeting orientation.” Items for rewards were my firm “rewards those who attend meetings,” “rewards those who lead meetings,” and “rewards those who organize meetings.” For strategic use, items were my firm “holds meetings to gather information,” “holds meetings to disseminate (share) information with attendees,” and “holds meetings to respond to (gathered) information.” Lastly, overuse was measured with the following items: my firm “has more meetings than what is required,” “has longer meetings than what is required,” and “holds meetings for meetings sake.” Participants responded to all items on a scale ranging from 1 (*strongly disagree*) to 5 (*strongly agree*). Hansen and Allen (2015) conducted a factor analysis of the scale and found that the four-factor solution fit the data best and explained 79% of the variability in the rotated sum of square factor loadings. Further, average variance extracted for each factor exceed 0.71 for all factors and Cronbach’s alpha was 0.79 or greater. In the current study, rewards (0.85), strategic use (0.67), and overuse (0.77) demonstrated acceptable internal consistency as assessed by Cronbach’s alpha, whereas the internal consistency of the policy focus measure was somewhat low (0.58).

##### Meeting and demographic variables

Participants reported on several factors of their last workplace meeting including meeting type, purpose (Allen et al., 2014), and number of attendees. Demographic variables included age, race/ethnicity, education level, job status, job tenure, and job level.

### Results

Descriptive statistics, alpha estimates of internal consistency, and correlations between study variables are included in Table 1. Hierarchical regression analyses were used to test each hypothesis. All hypotheses related to each outcome were tested concurrently in the same regression models.

**Table 1:** Descriptive statistics and correlations of focal variables in study 1.

Variable	*M*	*SD*	1	2	3	4	5	6	7
1. Meetings per week	3.37	3.82	-						
2. Rewards	2.71	0.87	0.02	(0.85)					
3. Strategic use	3.75	0.68	0.21*	0.39**	(0.67)				
4. Overuse	2.82	0.95	0.17*	0.08	0.12	(0.77)			
5. Policy	3.04	0.76	0.07	0.36**	0.45**	0.32**	(0.58)		
6. Team meeting effectiveness	3.65	0.67	0.09	0.22*	0.51**	-0.18*	0.17*	(0.83)	
7. Team meeting satisfaction	3.53	0.75	0.17*	0.26**	0.36**	-0.18*	0.09	0.48**	(0.85)

*N = 158. Diagonal values represent internal consistency estimates. ^∗^*p* < 0.05, ^∗∗^*p* < 0.001*.

#### Team Meeting Satisfaction

Hypotheses 1a and 2a,b predicted that overuse would be negatively related to team meeting effectiveness, whereas policy focus, rewards, and strategic use of group and team meetings would be positively related to team meeting satisfaction. In order to separate the influence of demographic factors on meeting satisfaction, the first step of the regression model included age, number of meetings attended per week, supervisory status, and job level as control variables, following best practice recommendations for statistical controls (Becker, 2005). Meeting load, or the number of meetings participants attend within a given period, has been demonstrated to affect employee job attitudes (Luong and Rogelberg, 2005). This step accounted for a significant amount of variance in meeting team satisfaction, *F*(4, 153) = 4.47, *p* = 0.002, *R*^2^ = 0.11.

In the second step of the analysis, the meeting orientation dimensions were jointly added to the model in order to test the relationships of interest and accounted for an additional 18% of variance in team meeting satisfaction, *F*(8, 149) = 7.46, *p* < 0.001. Results indicated that overuse (β = -0.20, *p* = 0.007) and strategic use of meetings (β = 0.36, *p* < 0.001) were significantly related to meeting satisfaction, thus providing support for hypotheses 1a and 2c. Policy focus (β = -0.01, *p* = 0.88) and rewards (β = 0.10, *p* = 0.18) were not related to meeting satisfaction so hypotheses 2a and 2b were not supported.

#### Team Meeting Effectiveness

The analytic strategy described for team meeting effectiveness as the outcome variable was followed to test hypotheses related to team meeting effectiveness. Hypothesis 1b predicted that overuse would be negatively related to team meeting effectiveness, and hypothesis 3a,c proposed that policy focus, rewards, and strategic use of meetings would be positively related to team meeting effectiveness.

As in the earlier test of meeting satisfaction, the first step of the regression model included age, number of meetings attended per week, supervisory status, and job level as control variables. These demographic variables did not account for a significant portion of the variability in meeting effectiveness, *F*(4, 156) = 0.72, *p* = 0.56, *R*^2^ = 0.02. The meeting orientation facets were then added to the model in the second step and explained an additional 29% of meeting effectiveness variance, *F*(8, 152) = 8.60, *p* < 0.001. Overuse (β = -0.22, *p* = 0.002) and strategic use of meetings (β = 0.53, *p* < 0.001) were significantly related to meeting effectiveness, which provided support for hypotheses 1b and 3c. Policy focus (β = -0.01, *p* = 0.89) and rewards (β = -0.01, *p* = 0.88) were not related to meeting satisfaction so hypotheses 3a and 3b were not supported. Complete results analyses are displayed in Table 2.

**Table 2:** Hierarchical multiple regression analyses predicting meeting satisfaction and meeting effectiveness in study 1.

	Meeting satisfaction		Meeting effectiveness	
Variable	Model 1	Model 2	Model 1	Model 2
*Controls*				
Age	0.24*	0.23*	0.03	0.02
Meetings/week	0.12	0.10	0.09	0.04
Supervisory status	-0.17	-0.22*	0.02	-0.05
Job level	-0.05	-0.10	0.09	0.02
*Focal variables*				
Policy focus		-0.01		-0.01
Rewards		0.10		-0.01
Strategic use		0.36**		0.53**
Overuse		-0.20*		-0.22**
*F*	4.47*	7.46**	0.72	8.60**
Adjusted *R*^2^	0.11	0.29	0.02	0.31
Δ*R*^2^		0.18		0.29

*Standardized regression coefficients are displayed. *N* = 158. ^∗^*p* < 0.05, ^∗∗^*p* < 0.001*.

## Study 2

The dimensions of meeting orientation may uniquely relate to employee work-related attitudes. According to Hansen and Allen’s (2015) theoretical propositions, meeting orientation should impact the culture, structure, and resources within an organization. Workplace meetings provide a setting in which supervisors and subordinates come together and interact in meaningful ways. Therefore, organizations with a high meeting orientation allow employees more opportunities for such meaningful interactions. High quality interactions are associated with trust, loyalty, respect, and obligation (Cropanzano and Mitchell, 2005). As a result, high quality leader-member exchange can result in organizational outcomes including: organizational commitment, turnover intentions, actual turnover, and job performance (Graen and Uhl-Bien, 1995).

However, certain facets of meeting orientation may be advantageous or disadvantageous relative to employee attitudes. For instance, employees who believe that their organization overuses group and team meetings—meeting overuse is a negative facet of meeting orientation that refers to the degree to which employees believe the organizations has too many meetings—may have poor work attitudes. Building from social exchange theory and POS theory, if employees believes that the organization does not value their time and wastes it on unnecessary group and team meetings, the employees are likely to have less favorable work attitudes. These positive (or negative) interactions may represent something beyond the dyadic relationship because leaders represent a proxy for the organization (Graen and Uhl-Bien, 1995). Subordinates who perceive their supervisors to be supportive may construe this interaction as an extension of the organization’s support. Through social exchange mechanisms, subordinates may further identify with the organization’s goals and care about organizational outcomes (Eisenberger et al., 1986). Therefore, we propose the following hypotheses:

Hypothesis 4: Overuse will be positively related to ITQ.
Hypothesis 5: Overuse will be negatively related to work engagement.

An organization’s emphasis on meeting orientation may contribute to both employee engagement and ITQ. Previous research demonstrated that employee engagement can be fostered in the context of workplace meetings (Allen and Rogelberg, 2013). Specifically, effectively managed group and team meetings create the conditions necessary for employees to engage in their work. Organizations with a stronger meeting orientation may provide employees with group and team meeting opportunities that assist with their ability to perform at optimal levels, connect with their role in the organization, and become fully immersed in their work (Bakker and Shaufeli, 2008).

In contrast, the group and team meeting context may also allow employees to engage in withdrawal behaviors—temporarily or permanently separating from their work roles (Harrison et al., 2006). For example, there are a variety of counterproductive team meeting behaviors that precipitously decrease employees’ attitudes related to their meetings and their organization overall (Lehmann-Willenbrock et al., 2016). As meetings are repeatedly held in contexts that are not conducive to the team’s best interests, individuals may feel drained and burned out since they are relying on this form of collaboration to facilitate the accomplishment of their goals. Thus, we believe that supervisors that exemplify the positive aspects of an organizations meeting orientation will enable engagement and reduce feelings related to quitting. The following are hypothesized:

Hypothesis 6: Policy focus (6a), rewards (6b), and strategic use of meetings (6c) will be negatively related to ITQ.
Hypothesis 7: Policy focus (7a), rewards (7b), and strategic use of meetings (6c) will be positively related to work engagement.

Although we expect that an organization’s meeting orientation is related to various job attitudes, such as ITQ and work engagement, additional team factors seem relevant in the context of this framework. That is, if meeting orientation is optimal or suboptimal, there are team factors that may strengthen positive job attitudes or reduce negative job attitudes. One good condition for teamwork, perceptions of voice, may promote good team behaviors (Gorden and Infante, 1991).

Voice refers to the degree in which employees feel as if they have voice and freedom to discuss their concerns (Gorden and Infante, 1991). Traditionally, this concept has been used as an important variable for employees who feel the need to change dissatisfying working conditions (Hirschman, 1970). Employees that perceive themselves to have a high voice may feel that: their ideas are valuable, they may share such ideas with others, and they may feel like they can actively participate in solving problems rather than simply acknowledging to decisions made by management (Gorden and Infante, 1991). In the context of meeting orientation, voice may serve as a resource that augments the effect of meeting orientation on positive workplace attitudes and depresses the effect of meeting orientation on negative workplace attitudes. In other words, we expect that the act of allowing dissenting views, ideas, or opinions in meetings may build a context of openness that empowers employees to take ownership of their work; in turn, this should promote feelings of engagement and reduce ITQ. Thus, we hypothesize:

Hypothesis 8: Voice in team meetings moderates the relationship between policy focus (8a) and strategic use of meetings (8b) and ITQ, such that the relationships will be more strongly negative when voice is low compared to high.
Hypothesis 9: Voice in team meetings moderates the relationship between policy focus (9a) and strategic use of meetings (9b) and engagement, such that the relationships will be more strongly positive when voice is high compared to low.

**Figure 2:**
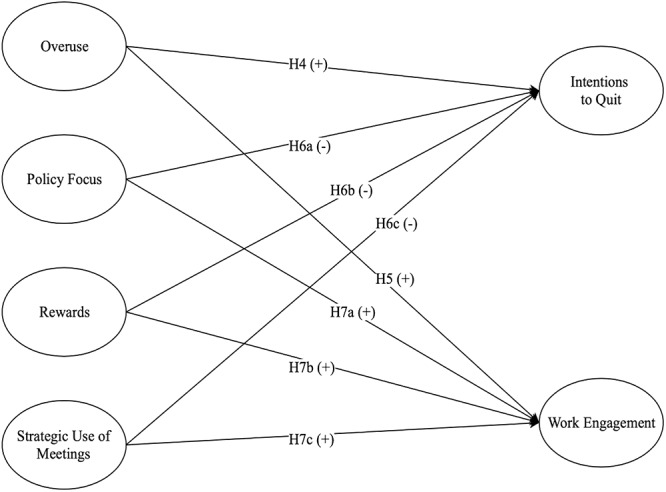
Hypothesized relationships in Study 2.

Figure 2 includes all hypothesized relationships tested in Study 2.

### Methods

#### Participants and Procedure

Participants in this study were recruited through a snowball sampling technique. Undergraduate students attending a large southeastern university enrolled in a psychology course were given a description of the study and Qualtrics link to share with full-time working adults in exchange for course extra credit. At the end of the survey, participants were encouraged to forward the survey link to other working adults who might be interested in participating. Participants were required to be employees in the United States who attend at least one work meeting per week. The sample consisted of 213 primarily White (66%) working adults, nearly split between males (48%) and females (52%).

#### Measures

##### Meeting orientation

The 12-item meeting orientation scale (Allen and Hansen, 2011) described in Study 1 was used in Study 2. Estimates of internal consistency as assessed by Cronbach’s alpha exceed 0.79 for all scales.

##### Work engagement

Employee work engagement was assessed using the Utrecht Work Engagement Scale (Schaufeli and Bakker, 2003). The scale consists of 17 items that measure three dimensions of work engagement: vigor, dedication, and absorption. Sample items include “At my work, I feel bursting with energy” (vigor), “I find the work that I do full of meaning and purpose” (dedication), and “I am immersed in my work” (absorption). Participants responded using a 7-point scale to indicate how often they feel each way at work from *never* to *always*. Engagement is typically examined as one factor due to high inter-correlations between the three dimensions (Allen and Rogelberg, 2013), as is the case in the present study. Internal consistency for this measure was 0.94.

##### Intentions to quit

A 3-item measure developed by Landau and Hammer (1986) was used to capture employees’ ITQ their work organization. Along a 7-point scale, participants reported the extent to which they agree with the statements (e.g., “I am actively looking for a job outside my current company”) from *not at all* to *extremely*. This measure demonstrated acceptable internal consistency with a Cronbach’s alpha of 0.88.

##### Voice

Voice was assessed using a 5-item measure from Gorden and Infante (1991) focusing on the degree to which employees felt they had voice and freedom to discuss concerns in their company or organization. Sample items included: “there was fear of expressing your true feelings on work issues” and “employees were penalized if they openly disagreed with management practices.” Ratings were made on a 7-point scale ranging from 1 (*never*) to 7 (*always*). Internal consistency for this measure was 0.75.

### Results

Descriptive statistics, alpha estimates of internal consistency, and correlations between study variables are included in Table 3. Hierarchical regression analyses were used to test each hypothesis, and complete results of the final models are displayed in Table 4.

**Table 3:** Descriptive statistics and correlations of focal variables in study 2.

Variable	*M*	*SD*	1	2	3	4	5	6	7	8
1. Meetings per week	2.69	2.90	-							
2. Reward	3.59	1.63	0.07	(0.91)						
3. Strategic use	5.04	1.31	0.16*	0.38**	(0.84)					
4. Overuse	3.87	1.62	0.26**	0.16*	0.12	(0.84)				
5. Policy	4.49	1.35	0.08	0.34**	0.53**	0.20*	(0.79)			
6. Voice	4.80	1.26	0.04	-0.08	0.16*	-0.35**	0.08	(0.75)		
7. Engagement	4.80	1.11	0.02	-0.15*	0.36**	-0.03	0.38**	0.22*	(0.94)	
8. Intention to quit	3.39	1.85	-0.01	-0.16*	-0.24*	0.23*	-0.30**	-0.44**	-0.48**	(0.88)

*N = 213. Diagonal values represent internal consistency estimates. ^∗^*p* < 0.05, ^∗∗^*p* < 0.01*.

**Table 4:** Hierarchical multiple regression analyses predicting intentions to quit and work engagement in study 2.

	Intentions to quit		Work engagement	
Variable	Model 1	Model 2	Model 1	Model 2
Meetings per week	-0.01	-0.02	-0.03	-0.03
Policy focus	-0.73**	-0.26**	0.18	0.27**
Rewards	-0.12	-0.11	0.01	0.01
Strategic use	-0.01	-0.56*	0.20*	0.24
Overuse	0.18*	0.19*	-0.05	-0.05
Voice	-0.78**	-0.89**	0.07	0.19
Voice x policy focus	0.66*	-	0.13	-
Voice x strategic use	-	0.83*	-	-0.06
*F*	13.29**	13.38**	7.68**	7.65**
Adjusted *R*^2^	0.29	0.29	0.18	0.18
Δ*R*^2^	0.02*	0.02*	0.01	<0.01

*N = 230. Standardized regression coefficients are displayed. *N* = 192. ^∗^*p* < 0.05, ^∗∗^*p* < 0.001. Δ*R*^*2*^ is from the model that included all variables aside from the interaction term*.

#### Intentions to Quit

Hypotheses 4 stated that overuse would be positively related to ITQ, whereas Hypotheses 6a,c proposed that policy focus, rewards, and strategic use of meetings would be negatively related to ITQ. Our control, number of meetings per week did not explain a significant amount of variability in ITQ, *F*(1, 211) = 0.02, *p* = 0.88, *R*^2^ = 0.00.

The meeting orientation facets were jointly added to the model in the second step and accounted for an additional 19% of variance in ITQ, *F*(5, 207) = 9.81, *p* < 0.05, *R*^2^ = 0.19. Overuse (β = 0.32, *p* < 0.001) and policy focus (β = -0.29, *p* < 0.05) were significantly related to ITQ, which supported Hypothesis 4 and 6a. Rewards (β = 0.07, *p* = 0.30) and strategic use of meetings (β = -0.08, *p* = 0.28) were not related to ITQ, which did not support Hypotheses 6b or 6c.

**Figure 3:**
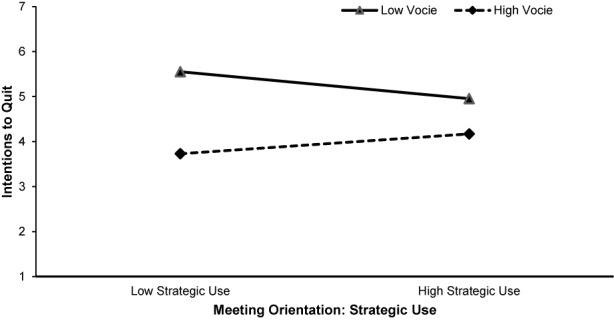
Strategic use of meetings interacted with voice such that using meetings strategically was most beneficial in reducing intentions to quit when voice was low (1 SD below the mean) compared to high (1 SD above the mean).

**Figure 4:**
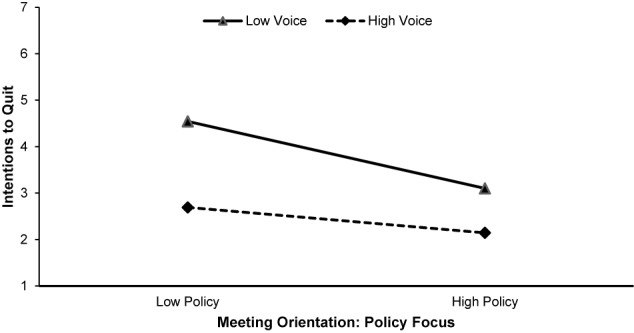
Policy focus interacted with voice such that the negative relationship between ITQ and policy focus was stronger when voice was low compared to high.

We also hypothesized that the relationship between policy focus and strategic use of meetings and ITQ would be moderated by voice, such that the relationships would be stronger when voice was high compared to low. First, we calculated an interaction term between policy and strategic use of meeting sand ITQ. For the regression analyses, the first step contained the control, number of meetings per week, the second step contained voice, the third step contained the four meeting orientations, and the interaction term was entered in the final step. The interaction term between policy and voice was significant and accounted for a significant portion of variance in ITQ, Δ*R*^2^ = 0.02, β = 0.66, *p* < 0.05, within the context of the entire model, *F*(7, 205) = 13.30, *p* < 0.05, *R*^2^ = 0.31, supporting Hypothesis 8a. Similarly, the interaction term between strategic use in meetings and voice was significant, Δ*R*^2^ = 0.02, β = 0.07, *p* < 0.05, within the context of the entire model, *F*(7, 205) = 13.38, *p* < 0.05, *R*^2^ = 0.31, supporting Hypothesis 8b. The interactions are depicted in Figure 3, 4.

#### Work Engagement

Hypotheses 5 proposed that overuse of meetings would be negatively related to work engagement, and Hypotheses 7a,c stated that policy focus, rewards, and strategic use of meetings would be positively associated with work engagement. The first step with the control variable, number of meetings per week, did not explain a significant amount of variance in work engagement, *F*(1, 211) = -0.08, *p* = 0.78, *R*^2^ = 0.00.

The four meeting orientation facets were added to the model in the second step and accounted for an additional 19% of variance in work engagement, *F*(5, 207) = 9.97, *p* < 0.05, *R*^2^ = 0.19. Policy (β = 0.28, *p* < 0.05) and strategic use of meetings (β = 0.23, *p* < 0.05) were significantly related to work engagement in the appropriate directions so Hypotheses 7a and 7c were supported. Overuse (β = -0.11, *p* = 0.09) and rewards (β = -0.01, *p* = 0.86), however, were not related to ITQ, which did not support Hypothesis 5 or 7b.

We also hypothesized that the relationship between policy focus and strategic use of meetings and engagement would be moderated by voice, such that the relationship would be stronger for those with greater policy focus or strategically focused orientations. First, we calculated an interaction term between policy and strategic use of meeting sand ITQ. For the regression analyses, the first step contained the control, number of meetings per week, the second step contained voice, the third step contained the four meeting orientations, and the interaction term was entered in the final step. The interaction term was not significant for either policy (Δ*R*^2^ = 0.00, β = 0.13, *p* = 0.70) or strategic use (Δ*R*^2^ = 0.00, β = -0.06, *p* = 0.88).

## General Discussion

This paper represents the first empirical investigation of the meeting orientation construct. As the first, exploratory step in a broader investigation of organizational meeting orientation, the results of this study confirm a series of hypotheses that relate facets of meeting orientation, policy focus, rewards, strategic use, and potential overuse, to perceived team meeting effectiveness and team meeting satisfaction as well as ITQ and work engagement. In Study 1 which included all variables, strategic use was positively related to perceived team meeting effectiveness and satisfaction; overuse, on the other hand, was negatively related to perceived team meeting effectiveness and satisfaction, whereas rewards and policy were not related to either outcome. Extending our findings from Study 1, we explored the extent to which an organization’s orientation toward meetings influences employee attitudes toward the organization. We found that employees in firms with a stronger, positive meeting orientation (defined as high on strategy, policy, and rewards and low on overuse) were more engaged in their work than employees in firms with a weak or negative meeting orientation. Policy, rewards, and strategic use were positively related to engagement, whereas meeting overuse was negatively related. Similarly, our findings indicate that meeting orientation is also related to employee ITQ. Greater meeting overuse was associated with higher turnover intentions, whereas strategic use of meetings was negatively related to ITQ.

In Study 2, we expanded our focus to an important variable related to group dynamics: perceived voice in meetings. Employees who believe they have high voice in meetings are more likely to speak up to voice their concerns, thoughts, and opinions during a group meeting context (Gorden and Infante, 1991). Indeed, we found that voice moderated the relationship between some facets of meeting orientation and ITQ. In general, a stronger organizational meeting orientation toward strategic use of team meetings for sharing, reacting to, and action upon information and having specific policies for the use of group and meetings was more beneficial to lower ITQ when voice was low compared to high. These findings illustrate that, in the absence of productive climates toward group interactions, factors specific to the organizational team meeting context can compensate, thereby leading to a more favorable employee attitude.

Despite the strong pattern of results linking aspects of meeting orientation to group and team meeting outcomes and employees’ work attitudes, several of our hypotheses were not supported. Controlling for number of meetings attended per week and the unique contribution of each facet of meeting orientation, policy focus and rewards explained unique variability only in work engagement. One reason for the relatively small contributions of these facets may be that these facets are more nebulous and less concrete than the others. For example, many organizations may not have specific policies that promote group and team meetings that employees can readily identify, meaning that the policy focus aspect of meeting orientation may not be useful or that the scale needs to be modified. Similarly, employees may have difficulty recalling specific rewards that their organizations offer to people who attend, lead, or organize team meetings.

### Theoretical Implications

The results of these studies have several implications. First, although the fact of being unstudied does not necessarily warrant research into a new area, this paper provided preliminary evidence that facets of organizational meeting orientation are related, and in some cases quite strongly, to important team meeting outcomes. For instance, prior research has demonstrated that satisfaction with meetings is a unique component of overall job satisfaction, even controlling for all traditional predictors of job satisfaction (Rogelberg et al., 2010). Across the two studies reported in this paper, organizational meeting orientation explained 33% of the variability in team meeting effectiveness, 20% of team meeting satisfaction, 19% of ITQ, and 19% of employee engagement. Much research on improving group and team meetings focuses on individual meeting practices, such as using an agenda, which may be helpful in improving the meetings of specific managers, but does not address meeting processes and procedures fostered at the organizational level.

Second, a variety of meeting scholars (cf. Allen et al., 2015) have suggested that technological advances in the workplace have nearly made informational meetings, or meetings in which people gather and exchange information, irrelevant, and that these irrelevant and unnecessary team meetings have contributed to the negative view of meetings in popular culture. The results of the study, however, indicate that people are more satisfied and believe that their group and team meetings are more effective when the organization supports and extensively utilizes information sharing in team meetings.

Third, group and team meetings may serve as an important tool which allows for the facilitation of employee-supervisor interactions; guided by an organizational meeting orientation, these exchanges can be advantageous and disadvantageous toward work attitudes. For instance, if an employee evaluates the dyadic relationship positively, they may construe the interactions as an extension of the organization’s support, thus, may be more motivated to accomplish work tasks (Eisenberger et al., 1986). However, if an employee feels as if their supervisor requires attendance to too many irrelevant team meetings, the employee may evaluate these interactions negatively, thus, engage in withdrawal behaviors (Allen and Rogelberg, 2013). The effects of these interactions may ripple across work attitudes.

### Practical Implications

Organizations may have various organizational-level orientations (e.g., market, customer, technology) meant to advance the topic of interest (Hansen and Allen, 2015). Although meeting orientation is not an overarching business aim like those previously mentioned, there are potentials for positive outcomes related to employee engagement, transfer of knowledge, and dynamic capabilities (i.e., response to change) as explained by Hansen and Allen (2015) in their theoretical framework. Being that policy and overuse meeting orientations are related to these job outcomes, there seem to be high costs associated with overuse and turnover intentions but gains related to policy and managerial support. Our findings warrant several managerial and organizational implications.

In terms of managerial implications, our findings suggest that meeting leaders have the discretion to capitalize on planning and leadership behaviors associated with the various meeting orientation dimensions. First, managers should consider whether it is necessary to schedule a team meeting; if the information can easily be shared through email or one-on-one conversations, managers should take advantage of these alternative forms of communication rather than holding pointless meetings. Second, when calling employees for a necessary group or team meeting, leaders should only invite people for which the content is relevant. For instance, rather than a manager calling their entire team, managers can make decisions as to which collaborators are essential to accomplish the meeting’s purpose. Third, to respect everyone’s time, meeting leaders should use an agenda as a roadmap to guide and end the team meeting when the items are completed. Fourth, it is crucial that meeting leaders utilize group and team meetings as a strategic tool to gather, disseminate, and respond to information relevant to all attendees.

In terms of organizational implications, our findings suggest that organizations can use meeting orientation as a competitive advantage to guide skills, behaviors, and processes of leaders and employees. First, organizations should assess where they fall within the four dimensions of meeting orientation; if necessary, organizations should make adjustments to the policies, procedures, and practices surrounding their meeting usage. Second, since group and team meetings may be perceived as interruptions from daily work tasks, organizational leaders should instruct on when it is appropriate to hold team meetings. Third, organizations should institute policies, procedures, or training programs to instruct managers on good team meeting practices (e.g., temporal, physical, cross-cultural considerations).

### Limitations

The findings of the study are an encouraging first step in the exploration of organizational level attitudes toward team meetings that can affect individual level outcomes, but a number of limitations must be considered when interpreting these findings. Most importantly, data examined in this study is cross-sectional in nature, which precludes drawing causal connections between variables, especially considering the scant literature and theorizing on meeting orientation generally. Furthermore, the cross-sectional, same-source data also makes the findings less potent. Although the models in this study depict meeting orientation leading to team meeting effectiveness, team meeting satisfaction, ITQ, and work engagement, it is entirely plausible that the opposite is true. For example, perhaps people who think their meetings are effective and satisfying believe that the organization strategically uses (and does not overuse) meetings. Future research should examine meeting orientation using a variety of data sources, such as objective, behaviorally based measures of team meeting effectiveness or quality, and relate these two ratings of meeting orientation.

Second, participants in this study represented a wide variety of organizations and were therefore each rating different organizations and different meetings. This is both a strength (i.e., increases generalizability) and limitation (i.e., hard to make specific predictions) of the studies. To strengthen the design, future research on meeting orientation should contain a combination of individual and organizational levels of analyses, such that multiple data points are collected within each organization to make comparisons across organizations possible. As meeting orientation is inherently an organizational level factor, of interest to meeting researchers should be how organizations with different meeting orientations conduct and approach group and team meetings, and another area that he may be how individuals with in those organi zations perceive their meetings.

Third, we implemented several strategies to mitigate concerns of common method variance given the cross-sectional nature of these studies (Podsakoff et al., 2003). To reduce demand characteristics and evaluation apprehension, we assured participants that their responses would remain anonymous and that there were no right or wrong answers. To mitigate order effects, priming effects, and item-context-induced mood states, we counterbalanced the measures and items through randomization (Podsakoff et al., 2003; Conway and Lance, 2010). To optimize comprehension, each item was simple, specific, and concise.

### Future Directions and Propositions for Teams Over Time

Although the forgoing studies substantiate the existence of meeting orientation, they cannot directly speak to how meeting orientation impacts teams at initial formation and over time as they work in the organization. However, an organization’s orientation toward the use of team meetings in each of the four facets could have implications for the ways in which teams develop and evolve over time. In our approach to meeting orientation, a “positive” orientation includes high levels of strategic use, policy focus, and rewards, whereas a negative orientation is low on those facets and high on overuse. Based on the findings reported in this paper, we develop several propositions below regarding meeting orientation. With respect to how teams develop over time, a positive meeting orientation may play an important role in establishing the working environment of new teams, acclimating new team members to the team and organization’s culture, fostering high-quality interactions with co-workers, enhancing commitment to the team and organization, and creating more stable team memberships.

Future research on team meeting orientation should focus on the measurement of full teams given that perceptions of meeting quality may be driven by the role held by the meeting participant (e.g., leader, attendee). Decades of organizational research have compared self, peer, and supervisor ratings on perceptions of traits, skills, abilities, and performance levels; at best, self-ratings demonstrate a moderate relationship to objective measures (Mabe and West, 1982; Harris and Schaubroeck, 1988; Bass and Yammarino, 1991). Team meetings may serve as another context in which there are discrepant ratings between roles, driven by various biases (e.g., Greenwald, 1980; Goethals, 1986). In fact, Cohen et al. (2011) noted that employees in higher positions of power tended to rate their meetings as higher quality compared to others. Perhaps these discrepant meeting perceptions are more complicated than a role differences but also a function of meeting type. For instance, status update meetings may be more valuable to the project manager than the attendees, however, a strategic planning meeting may be valuable to all attendees involved. Organizational leaders are often hiring new employees and launching new teams targeting projects of interest (Lester et al., 2002). Team comprised predominantly of new organizational members enter an environment where newcomer challenges exist (Chen and Klimoski, 2003), socialization to the organization is needed (Allen et al., 1999), and meeting orientation essentially defines how the team operates from a team meeting perspective. Given these challenges, it is likely that a positive meeting orientation as just defined would facilitate team performance generally, while a negative meeting orientation may hinder such progress in these newly formed and newly constituted teams. Further, over time, we anticipate that although team performance of new teams general improves with familiarity and codification of group processes, the stable meeting orientation (positive or negative) will create an artificial boundary condition on team performance either enabling maximal performance (i.e., positive meeting orientation) or constraining performance to a less than optimal level (i.e., negative meeting orientation). Thus, the following propositions are suggested:

Proposition 1a: Newly constituted teams will perform better in organizations with a positive compared to a negative meeting orientation.

Proposition 1b: Newly constituted teams performance will be optimized over time in an organization with a positive meeting orientation compared to a negative meeting orientation.

Team member change is one of the most common forms of changes in teams (Summers et al., 2012). Team member change can occur for a variety of reasons, but member change can often lead to, or be, a disruptive event (Olekalns et al., 2003). Member change has been conceptualized as a possible stimulant of team creativity as new members bring new ideas (Choi and Thompson, 2005), as a disruptive event that can lead to teams examining their processes and interaction strategies with an eye toward improvement (Zellmer-Bruhn, 2003), or as an opportunity for knowledge transfer and team functioning to decrease if core members change (Summers et al., 2012). We anticipate that team members will change less frequently as employees are less likely to think about quitting the organization entirely, and are more engaged in their work, when they perceive the organization to have a positive meeting orientation.

Proposition 2: Teams will experience less member change over time in organizations with a positive compared to a negative meeting orientation.

A critical role of meetings in team functioning is to act as a space for knowledge transfer among team members (Allen et al., 2014). Knowledge transfer includes passing information between individuals, groups, or organizations (Argote and Ingram, 2000), and knowledge/information sharing is a positive predictor of team performance (Mesmer-Magnus and DeChurch, 2009). As team members share information more frequently, the pool of information available for other team members to use increases, which can improve team performance (Hackman, 1987). When team meetings are used strategically and when necessary, teams may engage in increased information sharing behaviors, which may result in increased performance over time. Therefore, we propose:

Proposition 3: There is a positive a relationship between team information sharing over time and an organization’s meeting orientation.

## Conclusion

Unlike other organizational orientations (e.g., entrepreneurial), no empirical studies have investigated the consequences of meeting orientation. Studies 1 and 2 suggest that meeting orientation is related to individual perceptions of team meeting effectiveness, team meeting satisfaction, ITQ, and employee engagement even when controlling for several demographic variables. Although meeting orientation is not a predominant business aim, we see potential costs associated with meeting overuse but potential gains associated with strategic usage. Additionally, meeting orientation is an organizational level environmentally constraining construct with implications for new teams and for established teams. Over time, the meeting orientation of an organization has the potential to enable or constrain team performance and our hope is that the studies and propositions here will spur additional work by researchers on this important meeting science domain.

## Ethics Statement

The institutional review board (IRB) for the University of Nebraska Medical Center and the University of Nebraska at Omaha approved an exempt IRB protocol for the forgoing study. In this case, consent was given by participation in the surveys provided and completion of the survey was that consent and no identifying information was asked on the survey.

## Author Contributions

All authors listed have made a substantial, direct and intellectual contribution to the work, and approved it for publication.

## Conflict of Interest Statement

The authors declare that the research was conducted in the absence of any commercial or financial relationships that could be construed as a potential conflict of interest.
